# Sex-specific longitudinal changes in resting heart rate and all-cause heart failure: insights from the HUNT study

**DOI:** 10.3389/fcvm.2026.1752910

**Published:** 2026-03-18

**Authors:** Linda M. S. Hansen, Sumaya S. H. Jui, Tonje Braaten, Håvard Dalen, Lars B. Forr-Garnvik, Trine Karlsen

**Affiliations:** 1 Faculty of Nursing and Health Sciences, Nord University, Bodø/Levanger, Norway; 2 Department of Community Medicine, UiT the Arctic University of Norway, Tromsø, Norway; 3 Department of Circulation and Medical Imaging, Norwegian University of Science and Technology, Trondheim, Norway; 4 Department of Medicine, Levanger Hospital, Nord-Trøndelag Hospital Trust, Levanger, Norway; 5 Department of Cardiology and Cardiothoracic Surgery, St. Olavs Hospital, Trondheim University Hospital, Trondheim, Norway

**Keywords:** epidemiology, hazard ratio, heart failure, regression analysis, resting heart rate

## Abstract

**Aims:**

To investigate sex-specific associations between longitudinal resting heart rate (RHR) and new-onset heart failure (HF) using RHR change and RHR trajectories.

**Methods:**

Participants from the Trøndelag Health Study attending two or three surveys between 1995 and 2019 were included. We investigated the association between new-onset HF and RHR using RHR categories based on the standard deviation of baseline RHR (12 bpm), continuous RHR modeled using restricted cubic splines (*n* = 47,712; mean 12-year follow-up), and latent class trajectory models (*n* = 47,162; mean 7-year follow-up). Cox regression was used to estimate adjusted hazard ratios (HR) and 95% confidence intervals (95% CI).

**Results:**

During follow-up, 2,880 of the 47,712 participants developed HF. The HF incidence rate was lower in women than men (4.27 vs. 5.68 per 1,000 person-years; ratio (95% CI) 0.67 (0.57–0.77). Baseline RHR was 74 bpm in women and 70 bpm in men, and 74% maintained their RHR (±12 bpm) from baseline to the second attendance (mean change −2 ± 12 bpm). Each 10 bpm higher RHR was associated with higher HF risk for both women and men with HRs (95% CI) 1.15 (1.03–1.28) and 1.09 (1.00–1.20), respectively. Participants with a high RHR trajectory had higher HF risk than the low RHR trajectory with HRs (95% CI) 1.43 (1.14–1.79) for women and 1.41 (1.16–1.72) for men.

**Conclusion:**

All-cause HF was similarly associated with increased RHR and a high RHR trajectory for women and men. Estimating HF risk using RHR trajectories provided stronger associations than a single RHR measurement.

## Introduction

Heart failure (HF) affected approximately 64 million people globally in 2017 (1), with a similar lifetime risk for European women and men after the age of 75 years (2). Women generally develop HF 4–5 years later than men (2), and risk factors such as diabetes mellitus, hypertension, smoking, and obesity may have a greater impact on all-cause HF in women than in men (3). Additionally, hormonal and immune responses, pregnancy complications, and menopause may represent sex-specific risk factors for HF in women (4). Although sex differences in HF subtypes and risk factors are acknowledged in preventive guidelines (5), it is unclear if there are sex differences in potential prognostic markers like changes in resting heart rate (RHR).

RHR is an easy, non-invasive cardiac parameter that has previously been associated with both risk factors for HF and its incidence (6, 7). RHR is generally 3–7 bpm higher in women than in men and is also affected by age, genetics, and lifestyle factors such as physical activity and smoking (8). A normal RHR (e.g., 60–84 bpm) reflects a physically active lifestyle, whereas a high RHR (e.g., ≥85 bpm) may imply the presence of risk factors for all-cause HF (e.g., older age, hypertension, physical inactivity, and smoking) (7). In pooled sex analyses from two meta-analyses of cross-sectional studies, each 10-bpm higher RHR was associated with an 18%–19% higher relative risk of incident HF (6, 9). In a sex-specific analysis, men had a 14% higher relative risk of HF for each 10-bpm higher RHR, with no corresponding association observed in women (9). A single measurement of RHR can reflect physiological and environmental risk factors for HF, but it is currently unknown whether longitudinal RHR may better reflect long-term changes in cardiovascular physiology or pathophysiology (7).

Previous studies have shown that changes in RHR are associated with all-cause mortality and ischemic heart disease (10–12). Studies that investigated the associations between longitudinal RHR and HF did not exclude participants with pre-existing HF (13–15) or typically had short observation periods, with 3–5 years between RHR measurements (16, 17). One study reported a higher risk of all-cause incident HF for women than for men when comparing a high RHR trajectory (>72 bpm) with a low RHR trajectory (<60 bpm) (18). To our knowledge, no previous studies have investigated the longitudinal relationship between RHR and HF using an integrated approach combining categorical, continuous, and trajectory-based analyses. Moreover, long-term research on RHR trajectories and incident HF remains scarce in European populations. We therefore investigated sex-specific hazard ratios between longitudinal RHR and all-cause incident HF in a Norwegian adult cohort from the Trøndelag Health Study (HUNT), using both changes in RHR and RHR trajectories.

## Materials and methods

### Study design and population

**Figure 1:**
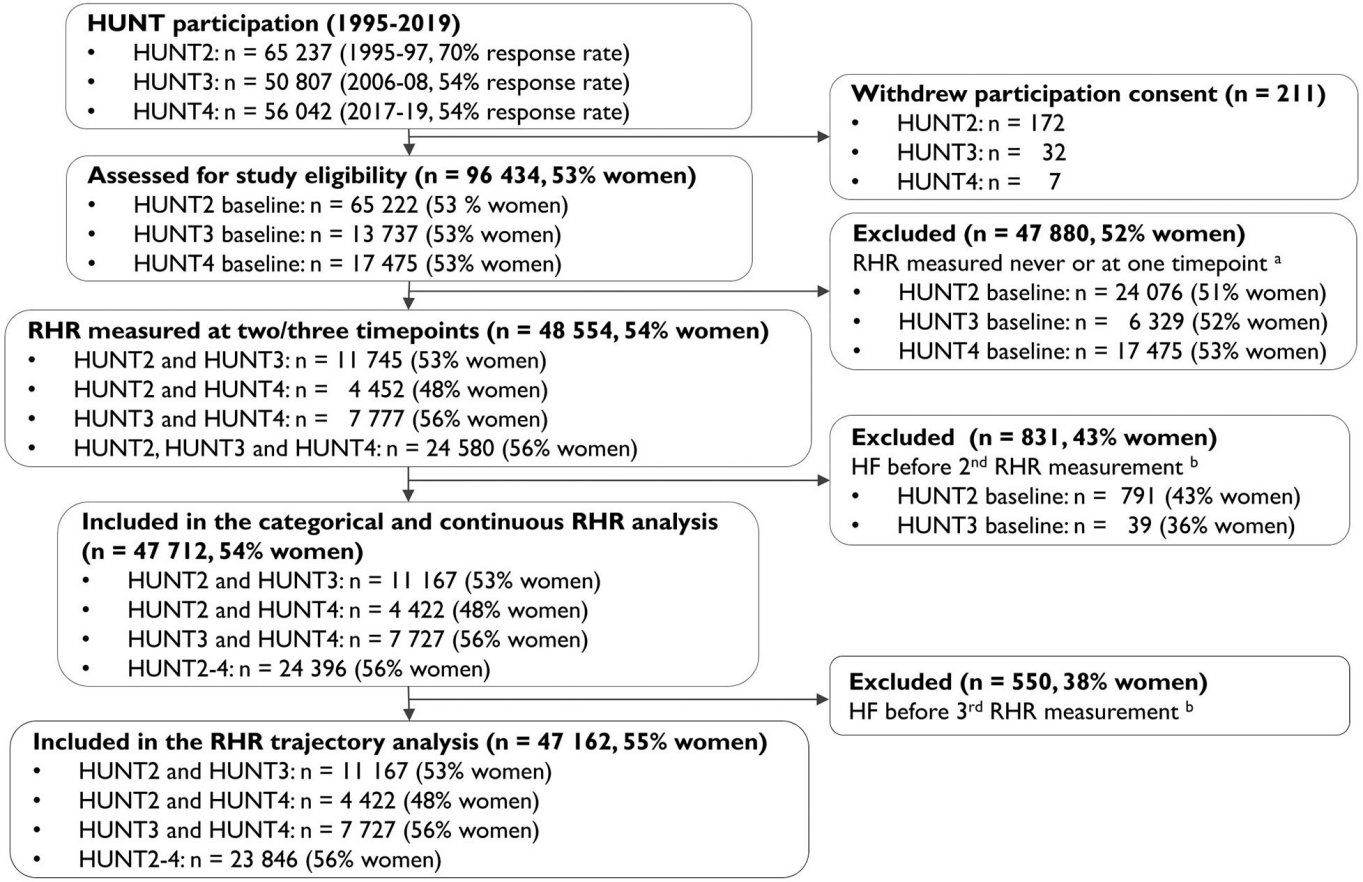
Flow of the participants included in the study. HUNT = The Trøndelag health study, RHR = resting heart rate, HF = heart failure, ^a^RHR set as missing if the participant was pregnant during clinical measurement, ^b^Incidence HF from hospital journals, the Norwegian Cause of Death Registry, or self-reported HF at baseline HUNT3.

The Trøndelag Health Study (HUNT) cohort invited all adult residents of Nord-Trøndelag County, Norway, to participate in four survey waves between 1984 and 2019 (19). Data from 1995 to 97 (HUNT2), 2006–08 (HUNT3), and 2017–19 (HUNT4) were used in this study. Briefly, the 96,436 individuals participating in HUNT2, HUNT3 and/or HUNT4 were above 20 years old, and 53% were women (19, 20). Participation rates were 70% in HUNT2% and 54% in both HUNT3 and HUNT4 (19). Details of the HUNT study design and population have been described elsewhere (19, 20). We excluded 47,880 participants (52% women) with fewer than two RHR measurements (50% of participants from HUNT2–4). For the categorical and continuous analyses, follow-up for incident HF started at the second RHR measurement; therefore, we excluded 831 participants (43% women) who were diagnosed with new-onset HF before the second RHR measurement (Figure 1). For the RHR trajectory analysis, we additionally excluded 550 participants (38% women) who were diagnosed with new-onset HF before the third RHR measurement (i.e., before completion of the trajectory period), as HF occurring during the trajectory period would introduce outcome-dependent dropout and potential reverse causation (21). Overall, we included participants aged 20–101 years. Of these, 47,712 (54% women) contributed to the longitudinal RHR vs. all-cause new-onset HF analyses, and 47,162 (55% women) contributed to the RHR trajectory vs. all-cause new-onset HF analyses.

This study was conducted in accordance with the Declaration of Helsinki and approved by the Regional Committee for Medical Research Ethics Central (2018/2416). All participants in HUNT provided written informed consent.

### Outcome

All-cause incident HF during follow-up was identified using data from the Nord-Trøndelag Hospital Trust (i.e., two local hospitals, including St. Olavs University Hospital) and the Norwegian Cause of Death Registry, based on the International Classification of Diseases (ICD-10 and ICD-9) codes I50 and 428. Diagnoses of HF, hypertensive heart disease, and cardiomyopathy recorded in hospital records from 369 participants were previously validated (22) according to current European Society of Cardiology guidelines (23).

### Clinical investigations

Clinical measurements and venous blood sampling in the HUNT investigations were performed by trained health professionals (19, 20). Biological material was stored in the HUNT Biobank (24). RHR and blood pressure were measured three times at 1-min intervals after a 2-min seated rest and before blood sampling, using an arm cuff and an automated oscillometric method (Dinamap 845XT analyzer, Critikon, Tampa, FL) (19, 20, 25). RHR measurements obtained during pregnancy were set to missing. The mean of the second and third RHR and blood pressure measurements from each HUNT survey was used in the analyses.

Non-fasting serum cholesterol was measured using a Hitachi 911 autoanalyzer (Hitachi, Mito, Japan). Hypertension was defined as systolic blood pressure ≥140 mmHg and/or diastolic blood pressure ≥90 mmHg and/or self-reported use of medication influencing heart rate or blood pressure. Diabetes mellitus was defined as fasting serum glucose ≥7.0 mmol/L (HUNT2–3), 2 h post-load serum glucose ≥11.1 mmol/L (HUNT4), or self-reported diabetes mellitus.

### Other covariates

All-cause incident atrial fibrillation was identified using hospital data from the Nord-Trøndelag Health Trust and the Norwegian Cause of Death Registry. The HUNT questionnaires contained self-reported data on leisure-time physical activity, educational level, smoking, cardiovascular diseases (i.e., stroke, angina pectoris, and myocardial infarction), use of medication for cardiovascular diseases, and treatment of hypertension and diabetes mellitus (20). Educational level was categorized into three levels: low (primary school, 7–10 years), middle (1–2 years of high school or university-qualifying examination), and high (university or other post-secondary education). Participants were categorized as current smokers if they reported daily or occasional smoking. Physical activity level was categorized as inactive, low, moderate, or high based on a previously published HUNT physical activity index (26). We estimated cardiorespiratory fitness as peak oxygen uptake using a HUNT-study-specific non-exercise regression prediction model based on age, physical activity level, RHR, and waist circumference (26).

### Statistical analysis

Baseline characteristics and variable distributions are presented as absolute numbers and percentages, means ± standard deviations for normally distributed continuous data, and medians with interquartile ranges (IQRs) for skewed data. Multiple imputation by chained equations was used to handle missing data, with twenty imputed datasets, stratified by sex. We considered the Missing at Random assumption reasonable because the probability of missingness could be accounted for by observed variables. The imputation model included predictors of both the incomplete variable and missingness itself, increasing the plausibility of this assumption (27). We verified the stability of inference across the imputed datasets by examining results from each imputation step (27). All analyses were performed using Stata for Windows, version 18.0 (StataCorp LLC, College Station, TX, USA).

In the categorical and continuous analyses, change in RHR was calculated as the difference in mean RHR between the first and second RHR measurements in HUNT2–4. The follow-up period extended from the second RHR measurement until the first all-cause new-onset HF, time of death from causes other than HF, or end of follow-up (30 March 2023), whichever came first. Non-HF deaths were censored at the date of death; participants who died from causes other than HF contributed follow-up time until death and were thereafter no longer considered at risk of incident HF events. We assessed the association with incident HF per 10 bpm change in RHR. Additionally, change in RHR between the first and second RHR measurements was categorized using a one-standard-deviation threshold based on the mean baseline RHR (12 bpm for both sexes), reflecting a substantial change relative to population variability (28) in the absence of established clinical cut-offs. Using this threshold, changes in RHR were classified as decreased, maintained, or increased. We modeled the association between continuous RHR and new-onset HF using restricted cubic splines with five knots positioned at Harrell's recommended percentiles (5th, 27.5th, 50th, 72.5th, and 95th), following the default implementation in Stata's *mkspline* command (29).

In the RHR trajectory analysis, we used latent class models to identify groups of RHR trajectories based on up to three RHR measurements, using the year of each HUNT survey as the timescale. We applied trajectory modeling as a descriptive approach to characterize distinct longitudinal patterns of RHR over time by grouping individuals with similar profiles, thereby providing a complementary perspective to analyses based on a single summary change measure. For each trajectory model, we evaluated polynomial specifications including intercept, linear, quadratic, and cubic terms. We used the Bayesian Information Criterion (BIC) to compare model fit across candidate trajectory models and required that each RHR trajectory included at least 5% of participants (30). The model providing the best fit and lowest BIC consisted of three sex-specific trajectory groups with up to quadratic polynomial terms. Models with four or more trajectories resulted in groups that were too small (i.e., <2.5% for women and <2.7% for men). Posterior predicted probabilities were used to estimate the probability of each participant belonging to a specific RHR trajectory group. Participants were assigned to one of the three trajectory groups based on the highest posterior probability. For participants who attended all three HUNT surveys, the follow-up period for new-onset HF began after the third RHR measurement time point used in the RHR trajectory analysis.

We used Cox proportional hazards models, stratified by sex, with attained age as the time variable, to estimate HRs and the 95% CIs for the association between changes in RHR and HF, and used Schoenfeld residuals to assess the proportional hazards assumption. Cox models were fitted in each imputed dataset and combined using Rubin's rules (31). Variables included in the analyses were chosen *a priori* based on factors that may affect both RHR and the risk of developing new-onset HF. Covariate adjustment was used to characterize conditional associations for prognostic purposes, rather than to estimate causal effects of intervening on RHR. Model 1 was adjusted for age. Model 2 was adjusted for age, physical activity, body mass index, smoking, and baseline RHR. Model 3 was adjusted for Model 2 covariates and hypertension (systolic blood pressure ≥140 mmHg, diastolic blood pressure ≥90 mmHg, or use of antihypertensive medication), diabetes (non-fasting serum glucose ≥11.1 mmol/L, HbA1c ≥11.1 mmol/L, or self-reported diabetes), educational level, and total cholesterol. Thus, Model 3 incorporates both clinical and lifestyle factors. Finally, Model 4 was adjusted for Model 3 covariates, atrial fibrillation, self-reported history of stroke, angina pectoris, myocardial infarction, and use of medication that may influence heart rate. We used time-updated values in all models. RHR trajectories were not adjusted for baseline RHR.

We performed sensitivity analyses in a subsample of participants with validated HF diagnoses from medical record files.

## Results

### Baseline characteristics

Table 1 presents baseline characteristics according to sex and RHR categories. Most women and men (74%) maintained their RHR, defined as being within one standard deviation of the first RHR measurement (±12 bpm). Women had a 4 bpm higher RHR and a 0.8–1.4 kg/m² lower BMI than men. Furthermore, a higher percentage of women were physically inactive compared to men, whereas a lower percentage of women had cardiovascular disease or hypertension. Women and men with a decreased RHR from baseline to the second RHR measurement had a 13–19 bpm higher mean baseline RHR and lower estimated VO₂_peak_ (1.4–4.2 mL·min^−1^) compared with participants with a maintained or increased RHR.

**Table 1:** Baseline participant characteristics.

Characteristic	Total	Decreased RHR (<−12 bpm)	Maintained RHR (±12 bpm)	Increased RHR (>12 bpm)
Women, *n* (%)	25,993 (54%)	4,420 (17%)	19,235 (74%)	2,338 (9%)
Age, years	44.6 ± 13.6	44.8 ± 14.1	44.5 ± 13.4	45.3 ± 14.3
Resting heart rate, bpm	73.8 ± 11.6	85.3 ± 11.5	72.0 ± 10.0	67.3 ± 10.1
SBP, mmHg (IQR)	125.0 (24.0)	130.0 (28.0)	124.0 (23.0)	125.0 (24.0)
DBP, mmHg	75.9 ± 11.5	79.2 ± 12.3	75.3 ± 11.2	75.3 ± 11.7
Body Mass Index, kg/m^2^ (IQR)	25.2 (5.4)	25.2 (6.0)	25.1 (5.3)	25.4 (5.4)
Total cholesterol, mmol/L	5.68 ± 1.26	5.76 ± 1.30	5.66 ± 1.25	5.69 ± 1.23
Smoking, *n* (%)	7,460 (29%)	1,406 (32%)	5,309 (28%)	745 (32%)
CVD^a^, *n* (%)	565 (2%)	89 (2%)	410 (2%)	67 (3%)
Hypertension^b^, *n* (%)	7,209 (28%)	1,664 (38%)	4,871 (25%)	674 (29%)
Diabetes^c^, *n* (%)	557 (2%)	123 (3%)	367 (2%)	67 (3%)
Physical activity level				
Inactive, *n* (%)	4,186 (18%)	660 (16%)	3,115 (18%)	411 (20%)
Low, *n* (%)	5,993 (25%)	1,030 (26%)	4,445 (25%)	518 (25%)
Moderate, (%)	6,044 (26%)	1,110 (28%)	4,430 (25%)	504 (25%)
High, (%)	7,396 (31%)	1,206 (30%)	5,574 (32%)	616 (30%)
Estimated VO_2peak_^d^, mL·min^−1^	29.2 ± 6.0	28.1 ± 6.0	29.5 ± 5.9	29.4 ± 6.6
Education				
Low (7–10 years primary school), *n* (%)	6,042 (24%)	1,121 (26%)	4,314 (23%)	607 (26%)
Middle (1–2 years high school), *n* (%)	11,039 (43%)	1,863 (43%)	8,202 (43%)	974 (42%)
High (University), *n* (%)	8,566 (33%)	1,353 (31%)	6,500 (34%)	713 (31%)
Men, *n* (%)	21,719 (46%)	3,195 (14%)	16,077 (74%)	2,447 (11%)
Age, years	44.9 ± 13.2	46.1 ± 13.5	44.7 ± 13.0	44.7 ± 13.9
Resting heart rate, bpm	69.6 ± 11.8	81.8 ± 11.5	68.2 ± 10.4	63.3 ± 10.3
SBP, mmHg (IQR)	134.0 (19.0)	138.0 (22.0)	133.0 (20.0)	133.0 (19.0)
DBP, mmHg	79.8 ± 11.4	83.2 ± 12.2	79.4 ± 11.1	78.3 ± 11.4
Body Mass Index, kg/m^2^ (IQR)	26.2 (4.2)	26.6 (4.5)	26.1 (4.1)	26.2 (4.4)
Total cholesterol, mmol/L	5.70 ± 1.13	5.88 ± 1.19	5.67 ± 1.12	5.61 ± 1.14
Smoking, *n* (%)	5,544 (26%)	964 (30%)	3,917 (25%)	663 (27%)
CVD^a^, *n* (%)	1,007 (5%)	156 (5%)	701 (4%)	150 (6%)
Hypertension^b^, *n* (%)	8,722 (40%)	1,648 (52%)	6,130 (38%)	944 (39%)
Diabetes^c^, *n* (%)	565 (3%)	114 (4%)	373 (2%)	78 (3%)
Physical activity level				
Inactive, *n* (%)	2,526 (13%)	419 (14%)	1,827 (13%)	280 (13%)
Low, *n* (%)	3,907 (20%)	602 (21%)	2,896 (20%)	409 (19%)
Moderate, (%)	4,959 (25%)	767 (27%)	3,673 (25%)	519 (24%)
High, (%)	8,224 (42%)	1,105 (38%)	6,191 (42%)	928 (43%)
Estimated VO_2peak_^d^, mL·min^−1^	38.1 ± 6.8	35.6 ± 6.7	38.4 ± 6.7	39.1 ± 7.3
Education				
Low (7–10 years primary school), *n* (%)	3,761 (17%)	644 (20%)	2,702 (17%)	415 (17%)
Middle (1–2 years high school), *n* (%)	11,291 (52%)	1,634 (52%)	8,369 (52%)	1,288 (53%)
High (University), *n* (%)	6,473 (30%)	883 (28%)	4,876 (31%)	714 (30%)

Baseline characteristics by change in resting heart rate [RHR, beats per minute (bpm)] from baseline to the 2nd measure. Values are presented as means (±SD), No. (percentages) or median [interquartile range (IQR)].

^a^ CVD: atrial fibrillation and self-reported stroke, angina pectoris, myocardial infarction, use of antiarrhythmic agents.

^b^ Hypertension: systolic blood pressure (SBP) ≥140 mmHg, diastolic blood pressure (DBP) ≥90 mmHg or use of medication influencing heart rate or blood pressure.

^c^ Diabetes: non-fasting serum glucose ≥11.1 mmol/L, HbA1c ≥11.1 mmol/L or self-reported diabetes in questionnaire.

^d^ VO_2peak_: peak oxygen consumption.

Women and men in the high RHR trajectory had a 15–30 bpm higher RHR and a 2.7–6.1 mL·min^−1^ lower estimated VO_2peak_ than those in the low or moderate RHR trajectories at baseline (Supplementary Table S1). Furthermore, a higher proportion of participants in the high RHR trajectory group had hypertension, a low educational level, and were current smokers compared with the low or moderate trajectory groups. For example, 47% of women and 59% of men in the high RHR trajectory group had hypertension, whereas the corresponding proportions in the low RHR trajectory group were 23% and 35%, respectively. Women were more likely than men to have a high baseline RHR, with 9% of women and 5% of men having an RHR ≥85 bpm (Supplementary Table S2). Moreover, a higher proportion of women and men with a high baseline RHR were current smokers, hypertensive, and physically inactive compared with participants with a low or normal baseline RHR.

### Longitudinal characteristics

The average observation period for RHR was 12.0 ± 3.2 years between baseline (HUNT2 or HUNT3) and the second RHR measurement (HUNT3 or HUNT4), and 10.6 ± 0.7 years between the second and third RHR measurements (HUNT3 to HUNT4). Of the 47,713 participants with a total of 586,542 person-years of follow-up (mean 12.3 years, maximum 16.5 years), 2,880 were diagnosed with new-onset HF (48% women; Table 2). Women with new-onset HF were diagnosed at an older age [median (IQR), 82 (74–88) years] than men [78 (69–84) years] and had a lower overall HF incidence rate (Table 2), with an incidence rate ratio of 0.67 (95% CI: 0.57–0.77).

**Table 2:** Change in resting heart rate and heart failure risk.

Characteristic	Per 10 bpm decreased RHR	Per 10 bpm increased RHR	Maintained RHR (±12 bpm)	Decreased RHR (<−12 bpm)	Increased RHR (>12 bpm)
Women					
Person-years	185,760	134,573	238,344	54,927	27,062
No. heart failure	802	567	931	289	149
Rate/1,000 person-years (95% CI)	4.32 (4.03–4.63)	4.21 (3.88–4.57)	3.91 (3.66–4.17)	5.26 (4.69–5.90)	5.51 (4.69–6.46)
Hazard ratio					
Model 1	1.13 (1.05–1.23)	1.20 (1.08–1.33)	1.00 (ref.)	1.24 (1.09–1.42)	1.27 (1.07–1.51)
Model 2	1.10 (1.01–1.20)	1.15 (1.04–1.28)	1.00 (ref.)	1.12 (0.97–1.30)	1.18 (0.99–1.41)
Model 3	1.06 (0.97–1.16)	1.15 (1.03–1.27)	1.00 (ref.)	1.09 (0.94–1.26)	1.17 (0.98–1.39)
Model 4	1.04 (0.95–1.14)	1.15 (1.03–1.28)	1.00 (ref.)	1.03 (0.89–1.20)	1.17 (0.98–1.39)
Men					
Person-years	141,424	124,781	198,468	38,739	28,997
No. heart failure	851	660	1,056	276	179
Rate/1,000 person-years (95% CI)	6.02 (5.63–6.44)	5.29 (4.90–5.71)	5.32 (5.01–5.65)	7.12 (6.33–8.02)	6.17 (5.33–7.15)
Hazard ratio					
Model 1	1.11 (1.03–1.20)	1.14 (1.04–1.25)	1.00 (ref.)	1.14 (1.00–1.30)	1.18 (1.01–1.38)
Model 2	1.05 (0.96–1.16)	1.13 (1.03–1.24)	1.00 (ref.)	1.02 (0.88–1.18)	1.13 (0.97–1.33)
Model 3	1.04 (0.95–1.13)	1.12 (1.02–1.23)	1.00 (ref.)	0.99 (0.85–1.14)	1.09 (0.93–1.28)
Model 4	1.00 (0.91–1.09)	1.09 (1.00–1.20)	1.00 (ref.)	0.93 (0.80–1.08)	1.07 (0.91–1.26)

Number of person-years and heart failures, rate/1,000 person years [95% confidence interval (CI)], and hazard ratios (95% CI) for all-cause heart failure in relation to change in resting heart rate [beats per minute (bpm), *n* = 47 712 (54% women)]. Model 1: adjusted for age. Model 2: adjusted for age, physical activity, body mass index, smoking and baseline RHR. Model 3: adjusted for Model 2 and hypertension (systolic blood pressure ≥140 mmHg, diastolic blood pressure ≥90 mmHg or use of antihypertensive medication), diabetes (non-fasting serum glucose ≥11.1 mmol/L, HbA1c ≥11.1 mmol/L, or self-reported), education and total cholesterol. Model 4: adjusted for Model 3, atrial fibrillation and time-updated self-reported history of stroke, angina pectoris, myocardial infarction and use of medication that influences heart rate.

**Figure 2:**
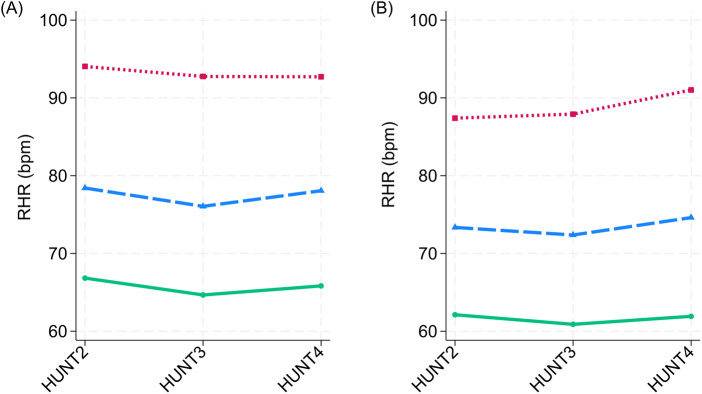
Resting heart rate trajectories in women **(A)** and men **(B)**, the Trøndelag health study, 1995–2019. Sex-specific means of resting heart rate (beats per minute) presented according to three HUNT surveys and three resting heart rate trajectory groups. Latent class models determined resting heart rate trajectory groups (Stata 18 traj).

Among the 47,162 participants with a total of 328,652 person-years of follow-up (mean 7.0 ± 4.1 years, maximum 16.5 years) included in the RHR trajectory analysis, 2,330 participants (50% women; Table 3) were diagnosed with new-onset HF. Women's RHR trajectories were consistently 3.7–4.8 bpm higher than men's (Figure 2). There was a nearly equal distribution of women and men in the low and moderate RHR trajectories, while 5% of women and 8% of men belonged to the high RHR trajectory (Table 3).

**Table 3:** Resting heart rate trajectories and heart failure risk.

Characteristic	Resting heart rate trajectories		
	Low	Moderate	High
Women	63.9–66.1 bpm	77.0–79.3 bpm	94.0–96.0 bpm
No. participants	12,353 (48%)	12,099 (47%)	1,333 (5%)
Person-years	84,122	83,150	9,351
No. heart failures	525	544	92
Rate/1,000 person-years (95% CI)	6.24 (5.73–6.80)	6.54 (6.02–7.12)	9.84 (8.02–12.07)
Hazard ratio			
Model 1	1.00 (ref.)	1.01 (0.90–1.14)	1.48 (1.19–1.85)
Model 2	1.00 (ref.)	0.96 (0.85–1.09)	1.34 (1.07–1.68)
Model 3	1.00 (ref.)	0.96 (0.85–1.09)	1.32 (1.06–1.65)
Model 4	1.00 (ref.)	1.00 (0.89–1.13)	1.43 (1.14–1.79)
Men	59.9–61.3 bpm	73.3–75.4 bpm	89.2–92.6 bpm
No. participants	9,511 (44%)	10,163 (48%)	1,704 (8%)
Person-years	65,772	73,747	12,510
No. heart failures	498	539	132
Rate/1,000 person-years (95% CI)	7.57 (6.93–8.27)	7.31 (6.72–7.95)	10.55 (8.90–12.51)
Hazard ratio			
Model 1	1.00 (ref.)	1.14 (1.01–1.29)	1.56 (1.28–1.89)
Model 2	1.00 (ref.)	1.07 (0.95–1.22)	1.42 (1.17–1.73)
Model 3	1.00 (ref.)	1.06 (0.93–1.20)	1.36 (1.12–1.66)
Model 4	1.00 (ref.)	1.08 (0.96–1.23)	1.41 (1.16–1.72)

Number of person-years and heart failures, rate/1,000 person years [95% confidence interval (CI)], and hazard ratios (95% CI) for all-cause heart failure in relation to trajectories groups of resting heart rate [beats per minute (bpm), *n* = 47 162 (55% women)]. Model 1: adjusted for age. Model 2: adjusted for age, physical activity, body mass index and smoking. Model 3: adjusted for Model 2 and hypertension (systolic blood pressure ≥140 mmHg, diastolic blood pressure ≥90 mmHg or use of antihypertensive medication), diabetes (non-fasting serum glucose ≥11.1 mmol/L, HbA1c ≥11.1 mmol/L, or self-reported), education and total cholesterol. Model 4: adjusted for Model 3, atrial fibrillation and time-updated self-reported history of stroke, angina pectoris, myocardial infarction and use of medication that influences heart rate.

Nelson–Aalen cumulative hazard estimates indicated a higher hazard of HF in participants older than 80 years compared with younger participants, and in men compared with women (Supplementary Figure S1). From baseline to the second RHR measurement, the overall mean change in RHR was −2 ± 12 bpm for women and −1 ± 12 bpm for men (Supplementary Figure S2). The maximum decrease in RHR from baseline to the second RHR measurement was −68 bpm for women and −67 bpm for men, and the maximum increase was 74 bpm for both sexes (Supplementary Figure S2). Nineteen participants (26% women) had an increase in RHR of 50 bpm or more.

### Categorical and continuous change in RHR and HF risk

In Model 4, women with an increase in RHR showed a trend toward a 17% higher risk of new-onset HF compared with women with a stable RHR (Table 2). No such trend was found for men. In continuous linear analyses of change in RHR in Model 4, each 10 bpm increase in RHR was associated with a 15% higher risk of HF in women and a 9% higher risk in men (Table 2). There was no association with a decrease in RHR for either sex, in either the categorical or the continuous analyses. Excluding 59 participants who were re-categorized from HF to non-valid HF in HUNT4HOPE did not affect the risk estimates (Supplementary Table S3).

**Figure 3:**
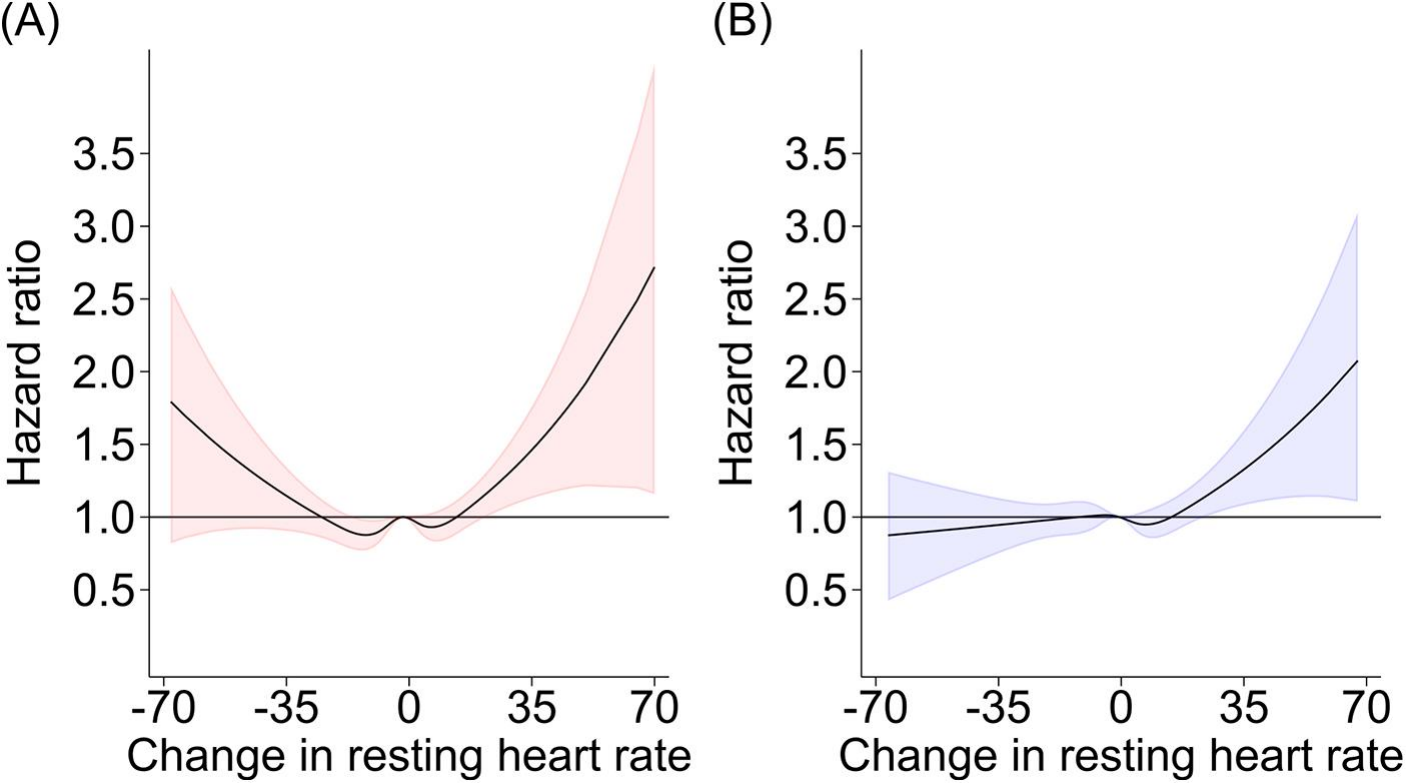
Restricted cubic splines displaying the association between resting heart rate and the risk of incidence of all-cause heart failure for women **(A)** and men **(B)**. Sex-specific hazard ratios and 95% confidence intervals presented according to change in resting heart rate (beats per minute) from baseline to follow-up. Restricted cubic splines with five knots were used to model the non-linear relationship between hazard ratio and change in resting heart rate (Stata 18 mkspline). Adjusted for age, physical activity, body mass index, smoking, hypertension (systolic blood pressure ≥140 mmHg, diastolic blood pressure ≥90 mmHg or use of antihypertensive medication), diabetes (non-fasting serum glucose ≥11.1 mmol/L, HbA1c ≥11.1 mmol/L, or self-reported), education, total cholesterol, atrial fibrillation and time-updated self-reported history of stroke, angina pectoris, myocardial infarction and use of medication that influence heart rate or blood pressure.

The restricted cubic splines displayed a non-linear relationship between RHR and HF in Model 4, indicating a higher risk of HF above a certain increase in RHR (e.g., above 25 bpm for women and 32 bpm for men; Figure 3). Although not significant for a decrease in RHR, the dose-response relationship appeared J-shaped for women, with no prominent J-shaped curve for men (Figure 3). Both sexes had wide confidence intervals for large changes in RHR (i.e., changes above 30 bpm).

Adjusting for lifestyle and known HF risk factors in Models 2 and 3 (e.g., hypertension, total cholesterol, and obesity) resulted in a 5%–7% reduction in risk estimates for women and a 1%–4% reduction for men per 10 bpm increase in RHR (Table 2). Further adjustment for cardiovascular diseases had a similar effect on the hazard ratios for both women and men, although women showed a slightly greater reduction in Models 2 and 3 (Table 2).

### RHR trajectories and HF risk

In Model 4, women and men in the high RHR trajectory had a 43% and 41% higher risk of new-onset HF, respectively, compared with the low RHR trajectory (Table 3). There was no association between the moderate RHR trajectory and HF compared with the low RHR trajectory. Adjusting for lifestyle factors had a similar impact on the hazard ratios for women and men with a high RHR trajectory (i.e., a 16%–20% risk reduction), whereas adjustment for known HF risk factors and cardiovascular diseases had a larger impact on the hazard ratios for women than for men (Table 3). Specifically, the risk for women increased from 32% in Model 3 to 43% in Model 4, while for men it increased from 36% to 41% (Table 3).

### Baseline HF risk

Women and men with a high baseline RHR (≥85 bpm) had a 19% and 23% higher risk of new-onset HF, respectively, compared with a normal baseline RHR (60–84 bpm). There was no association between a low baseline RHR (<60 bpm) and new-onset HF for either women or men (Supplementary Table S4). In continuous linear analyses, Schoenfeld residuals indicated a violation of the proportional hazards assumption for each 10 bpm increase in RHR when the baseline RHR was 50 bpm. There was no violation of the proportional hazards assumption when starting at 60 bpm. Each 10 bpm higher baseline RHR, starting from 60 bpm, was associated with a trend toward a 4% higher risk of new-onset HF for women and a statistically significant 9% higher risk for men in Model 4 (Supplementary Table S4).

## Discussion

In this study evaluating the association between longitudinal RHR and new-onset HF in a Norwegian cohort of 47,713 adults, the main findings indicated a similarly higher risk of HF in both women and men with an increased RHR or a high RHR trajectory. Each 10 bpm increase in RHR was associated with a 15% higher risk of HF for women and a 9% higher risk for men. We identified three sex-specific trajectory groups reflecting individual RHR patterns across up to 33 years of aging, with a 43% higher risk for women and a 41% higher risk for men with a high RHR trajectory compared with a low RHR trajectory. Our findings indicate that a large increase in RHR or a consistently high RHR may be early warning signs of HF for both sexes.

### Comparison with literature

#### Increase in RHR

The finding of a 15% higher risk of HF in women and 10% in men per 10-bpm increase in RHR aligns with two American studies (16, 17). However, our risk estimates were lower. One study reported a 13% (95% CI, 9%–16%) higher risk of HF per 5 bpm increase in RHR (16), while the other found a 38% (95% CI, 2%–86%) higher risk per 10 bpm increase (17) in combined analyses of both sexes. Differences in absolute risk, exclusion criteria, and methods of data collection may explain these discrepancies (16, 17). The incidence rate of HF in Norway is lower than in the USA (5.04–5.40 vs. 6.0–7.9 per 1,000 person-years) (32, 33), which aligns with the 6% of participants who developed HF in our study compared with the 18.7% reported in one American study (16). The other study (17) reported a 1.7% incidence of HF but may have introduced selection bias by excluding participants with any cardiovascular event prior to the final RHR measurement. Additionally, it assessed RHR via palpation (17), a method more prone to measurement error than the automatic oscillometric method (34) used in HUNT. Despite these differences, our findings support previous findings (16, 17) that increased RHR is associated with a similarly elevated risk of HF in women and men.

#### RHR trajectories

While we observed a 41%–43% higher risk of HF among both women and men with a high RHR trajectory, a US study reported a higher risk of HF for women (HR = 2.73; 95% CI, 1.51–4.95) than for men (HR = 2.37; 95% CI, 1.44–3.91) within the same trajectory (18). That study included 3,412 participants (54% women) free of known HF, with a mean age of 67 years at baseline, and followed participants for 10 years (18). Similar to our findings, 8.1% of participants developed HF during follow-up (18). Participants in our study may have had a more favorable baseline risk profile (5), as those in the American study were, on average, 23 years older, had a mean BMI that was 2.6 kg/m² higher, and 52% of women were hypertensive (18), compared with 28% in our study. In addition, we observed a higher proportion of participants with hypertension in the high RHR trajectory group compared with those in the low and moderate RHR trajectory groups. This finding may have clinical relevance, as it suggests that persistently elevated RHR could indicate underlying or insufficiently controlled hypertension, thereby potentially contributing to the progression of HF. Despite differences in baseline risk profiles, both studies identified a strong association between RHR trajectories and HF, underscoring the importance of longitudinal RHR as a biomarker for heart health.

#### Decrease in RHR

The absence of an association between a decrease in RHR and new-onset HF both aligns with and contrasts findings from previous studies (16, 17). The lack of statistical significance in the association between decreased RHR and HF aligns with a previous study observing RHR over five years (17). In a combined analysis using restricted cubic splines for women and men, a 20% lower risk of incident HF was observed with a decreased RHR (16). Variations in the covariates included in the analyses may explain the different findings between studies. Our study adjusted for HF risk factors such as total cholesterol, stroke, and angina pectoris (3), which the other study did not (16). Additionally, we used time-updated values for physical activity and smoking, rather than baseline values (16). Using time-updated values may affect risk estimates, as regular physical activity can lower RHR and reduce HF risk (8). For example, one study found a 91% higher risk of incident HF in unfit men compared with fit men (35). Further research is needed to understand potential clinical implications of decreased RHR.

#### Single time-point RHR vs. longitudinal RHR

A small proportion of participants, i.e., 5% of women and 8% of men, had an increased risk of HF associated with up to 30 years of elevated RHR. We found a stronger association between longitudinal RHR and incident HF than with baseline RHR data alone, suggesting that repeated RHR measurements may be particularly important in individuals with consistently elevated RHR for improved HF risk assessment and prevention. While a single RHR measurement can reflect underlying cardiovascular pathology, repeated measurements can capture long-term patterns and changes in physiological and environmental risk factors for HF (7). Additionally, RHR trajectories provide more information by incorporating a third RHR measurement into the analysis and allowing for longer exposure time. With more data points, a 33-year observation period, and older participants at the start of follow-up, we obtained higher risk estimates for women and men than when using only two RHR measurements. HF often develops gradually in both sexes due to cumulative exposure to risk factors (3), and including multiple RHR measurements offers a comprehensive view of individual RHR trends over time.

### Incidence rates and risk factors for HF

In our study, the incidence rate of HF per 1,000 person-years was 5.0 for women and 6.8 for men. These rates align with findings from a study of German men (36) but are higher than the overall estimate from 12 European countries, which was 3.2 per 1,000 person-years (37). Potential mechanisms for the lower incidence rate of HF in women compared to men include differences in risk factors such as age, diabetes mellitus, and hypertension (3). European women have been shown to develop HF 4–6 years later than men (2, 38), a finding confirmed in our study, where the median age at HF diagnosis was 84 years for women and 80 years for men. Aging is related to physiological changes in heart structure, such as increased left ventricular wall thickness and decreased left ventricular mass, and these changes might be sex-specific (39). Additionally, a recent European study found that hypertension, total cholesterol, and obesity contributed more to HF risk in women than in men (40). For example, a study reported that women with diabetes mellitus had a five-fold higher risk of HF compared with a two-fold risk in men, and women with hypertension had a three-fold higher risk compared with a two-fold risk for men (3). The risk-factor adjustments in our models revealed a consistent pattern, showing that known HF risk factors such as hypertension, total cholesterol, and obesity had a greater impact in women than in men. Overall, the lower incidence rate of HF in women compared to men in our study may be attributed to both biological differences and diverse risk factor profiles.

### Strengths and limitations

The main strengths of this study include a well-characterized, large cohort of 47,713 participants and a rigorous methodology that combines categorical, continuous, and trajectory-based analyses supported by comprehensive data on key heart failure risk factors (e.g., physical activity, blood pressure, and cardiovascular diseases). Importantly, this research provides long-term evidence on RHR trajectories and incident HF within a European population, where such studies have been notably scarce. The equal representation of women and men enabled sex-specific analyses, provided high statistical power, and minimized the risk of type II errors (41). The HUNT Study facilitates longitudinal comparisons through repeated clinical measurements, with questionnaires covering various topics and consistently maintaining items across surveys (19). Additionally, the intervals between RHR measurements in our study were 7–9 years longer than in previous studies (16, 17), enabling the use of more recent, time-updated values for RHR as well as potential confounders in our analysis.

Potential collider bias is a recognized concern in epidemiological studies, particularly for causal inference (21). Accordingly, we present sequentially adjusted models for transparency and to characterize conditional rather than causal associations. While Models 1–3 avoid conditioning on downstream cardiovascular disease, Model 4 additionally adjusts for established cardiovascular disease and heart-rate–affecting medications to evaluate prognostic associations, which may be susceptible to collider bias if interpreted causally. Requiring two RHR measurements resulted in the exclusion of 50% of otherwise eligible participants, which may have introduced selection bias and limited generalizability if follow-up participation systematically was related to baseline health status or cardiovascular risk. Misclassification of HF is a potential limitation (41); however, linkage to national health registers and high-quality hospital records substantially reduces this risk. HF was confirmed in 310 of the 369 participants evaluated in the HUNT4HOPE study and exclusion of non-validated HF cases did not alter risk estimates. We did not formally compare model fit, discrimination, or calibration between trajectory-based exposure and continuous RHR change. Our use of trajectory modeling was intended to provide a descriptive view of long-term patterns rather than to enhance risk prediction. HF is a heterogeneous condition with etiologies that may differ by sex (3). Because information on HF phenotype was unavailable and the cohort was investigated during a period of evolving diagnostic guidelines for diastolic dysfunction, the potential impact of underdiagnosed diastolic dysfunction in the cohort remains unknown. Thus, it remains unclear whether elevated RHR reflects physiological stimulation, such as increased sympathetic load, or an early warning sign of pathological remodeling, characterized by a significant decrease in stroke volume and a compensatory increase in RHR to maintain adequate cardiac output.

## Conclusion

All-cause HF was similarly associated with increased RHR and a high RHR trajectory in women and men. Estimating HF risk using RHR trajectories provided a stronger association between RHR and HF compared with using a single RHR measurement.

### Clinical implications

Repeated RHR measurements have the potential to be used more extensively as a low-cost clinical tool for monitoring heart disease risk over decades. Knowledge of longitudinal RHR might improve prevention strategies or initiate relevant treatments in high-risk individuals. With the advent of new and more accurate wrist-worn devices for measuring heart rate, the potential for home monitoring of high-risk individuals is substantial and may reduce the patient load on health services. Repeated RHR measurements might also provide valuable prognostic insights by minimizing the impact of temporary heart rate fluctuations. These measurements can help identify the small proportion of men and women in a population who have consistently elevated RHR and a high risk of developing HF. Future studies, as well as clinical investigations, should explore whether the underlying cause of elevated RHR is physiological or pathological. Additionally, long-term RHR measurements can reveal changes in HF risk profiles that might not be detectable with a single RHR measurement and highlight individuals with high RHR who should be further evaluated.

## Data Availability

The data analyzed in this study is subject to the following licenses/restrictions: The Trøndelag Health Study (HUNT) has invited persons aged 13–100 years to four surveys between 1984 and 2019. Comprehensive data from more than 140,000 persons having participated at least once and biological material from 78,000 persons are collected. The data are stored in HUNT databank and biological material in HUNT biobank. HUNT Research Centre has permission from the Norwegian Data Inspectorate to store and handle these data. The key identification in the data base is the personal identification number given to all Norwegians at birth or immigration, whilst de-identified data are sent to researchers upon approval of a research protocol by the Regional Ethical Committee and HUNT Research Centre. To protect participants' privacy, HUNT Research Centre aims to limit storage of data outside HUNT databank, and cannot deposit data in open repositories. HUNT databank has precise information on all data exported to different projects and are able to reproduce these on request. There are no restrictions regarding data export given approval of applications to HUNT Research Centre. For more information see: http://www.ntnu.edu/hunt/data. Requests to access these datasets should be directed to kontakt@hunt.ntnu.no.

## Supplementary material

SUPPLEMENTARY FIGURE S1 Nelson-Aalen cumulative hazard estimates of heart failure. Sex-specific cumulative hazard presented according to age (years) for women (A, solid red line) and men (B, dotted blue line).

SUPPLEMENTARY FIGURE S2 Density plot for change in resting heart rate from baseline to follow-up. Sex-specific density presented according to change in resting heart rate (beats per minute) across a median of 12.2 years of aging for women (solid line) and men (dotted line).
