# Supplementary figures and images for: Sex-specific longitudinal changes in resting heart rate and all-cause heart failure: insights from the HUNT study
Source: Front Cardiovasc Med. 2026 Mar 18;13:1752910. doi: 10.3389/fcvm.2026.1752910 (PMC13038512; doi:10.3389/fcvm.2026.1752910)

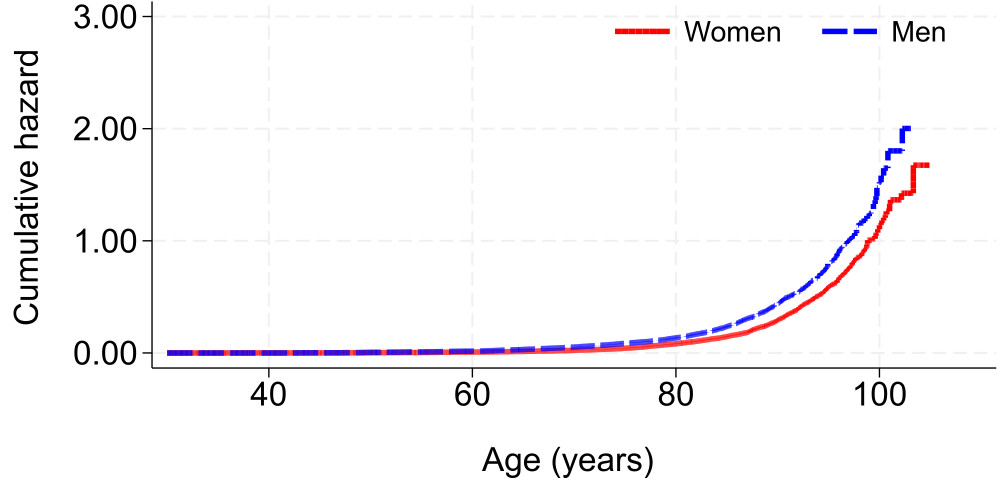

Supplement: SUPPLEMENTARY FIGURE S1 — Nelson-Aalen cumulative hazard estimates of heart failure. Sex-specific cumulative hazard presented according to age (years) for women (A, solid red line) and men (B, dotted blue line). [file Image1.jpeg]

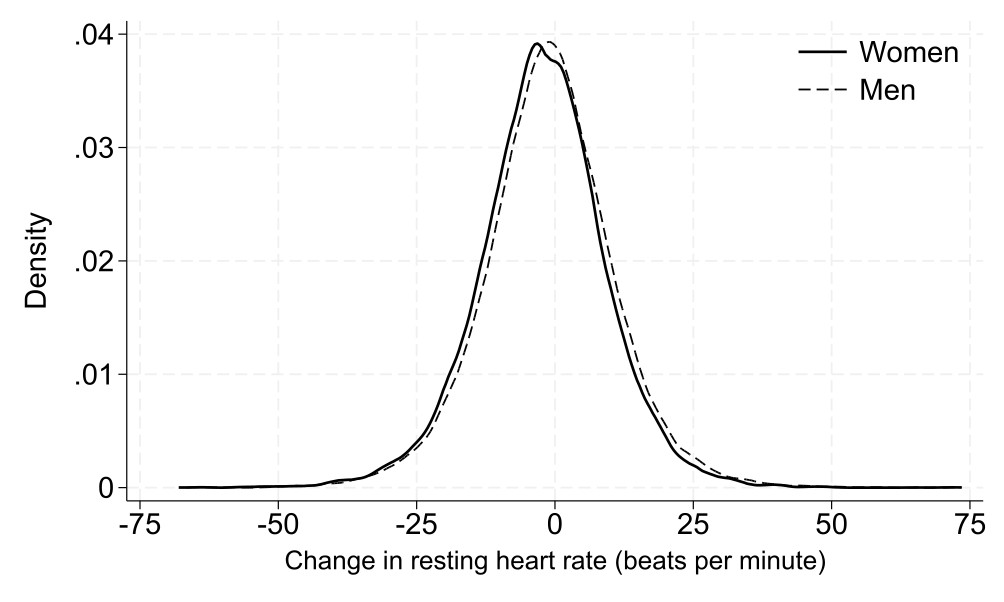

Supplement: SUPPLEMENTARY FIGURE S2 — Density plot for change in resting heart rate from baseline to follow-up. Sex-specific density presented according to change in resting heart rate (beats per minute) across a median of 12.2 years of aging for women (solid line) and men (dotted line). [file Image2.jpeg]
