# Supplementary material for: Sex-specific longitudinal changes in resting heart rate and all-cause heart failure: insights from the HUNT study
Source: Front Cardiovasc Med. 2026 Mar 18;13:1752910. doi: 10.3389/fcvm.2026.1752910 (PMC13038512; doi:10.3389/fcvm.2026.1752910)
Supplement: Supplementary file 3 [file Datasheet1.docx]

| **Supplementary data, Table S1.** Baseline participant characteristics by resting heart rate trajectories. | | | | |  |
| --- | --- | --- | --- | --- | --- |
|  | Total | Low RHR trajectory | Moderate RHR trajectory | High RHR trajectory | |
| **Women (n)** | 25785 (55%) | 12353 (48%) | 12099 (47%) | 1333 (5%) | |
| Age, years | 44.5 ± 13.6 | 44.5 ± 13.4 | 44.5 ± 13.8 | 45.4 ± 14.0 | |
| Resting heart rate, bpm | 73.8 ± 11.6 | 66.1 ± 7.4 | 79.3 ± 8.4 | 96.0 ± 11.8 | |
| SBP, mmHg (IQR) | 125.0 (23.0) | 122.0 (22.0) | 127.0 (23.0) | 135.0 (29.0) | |
| DBP, mmHg | 75.9 ± 11.5 | 73.6 ± 11.0 | 77.5 ± 11.4 | 82.4 ± 12.0 | |
| Body Mass Index, kg/m^2^ (IQR) | 25.1 (5.4) | 24.9 (5.0) | 25.3 (5.7) | 25.7 (6.3) | |
| Total cholesterol, mmol/L | 5.67 ± 1.26 | 5.60 ± 1.25 | 5.73 ± 1.26 | 5.89 ± 1.29 | |
| Smoking, n (%) | 7398 (29%) | 2871 (23%) | 4015 (33%) | 512 (39%) | |
| CVD ^a^, n (%) | 552 (2%) | 333 (3%) | 195 (2%) | 19 (1%) | |
| Hypertension ^b^, n (%) | 7095 (28%) | 2864 (23%) | 3609 (30%) | 622 (47%) | |
| Diabetes ^c^, n (%) | 553 (2%) | 214 (2%) | 294 (2%) | 45 (3%) | |
| Physical activity level |  |  |  |  | |
| Inactive, n (%) | 4142 (18%) | 1797 (16%) | 2109 (19%) | 236 (20%) | |
| Low, n (%) | 5943 (25%) | 2658 (23%) | 2938 (27%) | 347 (30%) | |
| Moderate, (%) | 5993 (26%) | 2992 (26%) | 2719 (25%) | 282 (24%) | |
| High, (%) | 7344 (31%) | 3950 (35%) | 3087 (28%) | 307 (26%) | |
| Estimated VO_2peak_ ^d^, mL·min^−1^ | 29.3 ± 6.0 | 30.5 ± 5.8 | 28.3 ± 5.9 | 25.8 ± 6.2 | |
| Education |  |  |  |  | |
| Low (7-10 years primary school), n (%) | 5953 (23%) | 2551 (21%) | 2979 (25%) | 423 (32%) | |
| Middle (1-2 years high school), n (%) | 10953 (43%) | 4958 (41%) | 5425 (45%) | 570 (44%) | |
| High (University), n (%) | 8533 (34%) | 4690 (38%) | 3527 (30%) | 316 (24%) | |
|  |  |  |  |  | |
| **Men (n)** | 21378 (45%) | 9511 (44%) | 10163 (48%) | 1704 (8%) | |
| Age, years | 44.7 ± 13.2 | 45.2 ± 13.3 | 44.2 ± 13.1 | 45.3 ± 13.1 | |
| Resting heart rate, bpm | 69.6 ± 11.8 | 61.2 ± 7.4 | 74.3 ± 8.4 | 89.2 ± 10.7 | |
| SBP, mmHg (IQR) | 134.0 (19.0) | 132.0 (19.0) | 134.0 (20.0) | 140.0 (22.0) | |
| DBP, mmHg | 79.7 ± 11.4 | 77.6 ± 10.9 | 80.7 ± 11.4 | 85.7 ± 11.5 | |
| Body Mass Index, kg/m^2^ (IQR) | 26.1 (4.2) | 25.8 (3.7) | 26.4 (4.4) | 26.9 (5.0) | |
| Total cholesterol, mmol/L | 5.69 ± 1.13 | 5.61 ± 1.12 | 5.72 ± 1.14 | 5.91 ± 1.17 | |
| Smoking, n (%) | 5454 (26%) | 1845 (20%) | 2981 (30%) | 628 (37%) | |
| CVD ^a^, n (%) | 963 (5%) | 552 (6%) | 357 (4%) | 54 (3%) | |
| Hypertension ^b^, n (%) | 8534 (40%) | 3285 (35%) | 4239 (42%) | 1010 (59%) | |
| Diabetes ^c^, n (%) | 557 (3%) | 200 (2%) | 274 (3%) | 83 (5%) | |
| Physical activity level |  |  |  |  | |
| Inactive, n (%) | 2480 (13%) | 907 (10%) | 1326 (15%) | 247 (16%) | |
| Low, n (%) | 3839 (20%) | 1498 (17%) | 1976 (22%) | 365 (24%) | |
| Moderate, (%) | 4881 (25%) | 2126 (24%) | 2333 (26%) | 422 (28%) | |
| High, (%) | 8094 (42%) | 4236 (48%) | 3394 (38%) | 464 (31%) | |
| Estimated VO_2peak_ ^d^, mL·min^−1^ | 38.1 ± 6.8 | 39.8 ± 6.6 | 37.1 ± 6.6 | 33.9 ± 6.6 | |
| Education |  |  |  |  | |
| Low (7-10 years primary school), n (%) | 3677 (17%) | 1483 (16%) | 1838 (18%) | 356 (21%) | |
| Middle (1-2 years high school), n (%) | 11114 (52%) | 4674 (50%) | 5504 (55%) | 936 (56%) | |
| High (University), n (%) | 6392 (30%) | 3276 (35%) | 2728 (27%) | 388 (23%) | |
| Values are presented as means (± SD), No. (percentages) or median (interquartile range). ^a^ CVD: self-reported stroke, angina pectoris, myocardial infarction, use of antiarrhythmic agents. ^b^ Hypertension: systolic blood pressure (SBP) ≥ 140 mmHg, diastolic blood pressure (DBP) ≥ 90 mmHg or use of medication that influences heart rate or blood pressure. ^c^ Diabetes: non-fasting serum glucose ≥11.1 mmol/L, HbA1c ≥11.1 mmol/L or self-reported diabetes in questionnaire. ^d^ VO_2peak_: estimated peak oxygen consumption. | | | | |  |

| **Supplementary data, Table S2.** Baseline participant characteristics by categories of baseline RHR. | | | | |  |
| --- | --- | --- | --- | --- | --- |
|  | Total | Low RHR  (< 60 bpm) | Normal RHR  (60-84 bpm) | High RHR  (≥ 85 bpm) | |
| **Women (n)** | 25993 (54%) | 2427 (9%) | 19305 (75%) | 4261 (16%) | |
| Age, years | 44.6 ± 13.6 | 45.4 ± 13.5 | 44.6 ± 13.5 | 44.3 ± 14.0 | |
| Resting heart rate, bpm | 73.8 ± 11.6 | 55.5 ± 3.5 | 72.0 ± 6.5 | 92.7 ± 7.8 | |
| SBP, mmHg (IQR) | 125.0 (24.0) | 121.0 (22.0) | 124.0 (23.0) | 132.0 (26.5) | |
| DBP, mmHg | 75.9 ± 11.5 | 71.5 ± 11.1 | 75.4 ± 11.1 | 80.9 ± 11.9 | |
| Body Mass Index, kg/m^2^ (IQR) | 25.2 (5.4) | 24.9 (4.8) | 25.1 (5.3) | 25.5 (6.3) | |
| Total cholesterol, mmol/L | 5.68 ± 1.26 | 5.52 ± 1.22 | 5.67 ± 1.25 | 5.81 ± 1.30 | |
| Smoking, n (%) | 7460 (29%) | 479 (20%) | 5405 (28%) | 1576 (37%) | |
| CVD ^a^, n (%) | 567 (2%) | 121 (5%) | 382 (2%) | 64 (2%) | |
| Hypertension ^b^, n (%) | 7209 (28%) | 591 (24%) | 4899 (25%) | 1719 (40%) | |
| Diabetes ^c^, n (%) | 557 (2%) | 44 (2%) | 385 (2%) | 128 (3%) | |
| Physical activity level |  |  |  |  | |
| Inactive, n (%) | 4186 (18%) | 326 (15%) | 3126 (18%) | 734 (19%) | |
| Low, n (%) | 5993 (25%) | 438 (20%) | 4484 (25%) | 1071 (28%) | |
| Moderate, (%) | 6044 (26%) | 573 (26%) | 4486 (26%) | 985 (26%) | |
| High, (%) | 7396 (31%) | 890 (40%) | 5492 (31%) | 1014 (27%) | |
| Estimated VO_2peak_ ^d^, mL·min^−1^ | 29.2 ± 6.0 | 31.7 ± 6.0 | 29.4 ± 5.8 | 26.9 ± 6.0 | |
| Education |  |  |  |  | |
| Low (7-10 years primary school), n (%) | 6042 (24%) | 493 (21%) | 4358 (23%) | 1191 (28%) | |
| Middle (1-2 years high school), n (%) | 11039 (43%) | 923 (39%) | 8249 (43%) | 1867 (44%) | |
| High (University), n (%) | 8566 (33%) | 981 (41%) | 6445 (34%) | 1140 (27%) | |
|  |  |  |  |  | |
| **Men (n)** | 21719 (46%) | 4333 (20%) | 15057 (69%) | 2329 (11%) | |
| Age, years | 44.9 ± 13.2 | 45.4 ± 13.4 | 44.6 ± 13.1 | 45.6 ± 13.1 | |
| Resting heart rate, bpm | 69.6 ± 11.8 | 54.4 ± 4.2 | 70.5 ± 6.5 | 92.3 ± 7.0 | |
| SBP, mmHg (IQR) | 134.0 (19.0) | 131.0 (19.0) | 134.0 (19.0) | 140.0 (22.0) | |
| DBP, mmHg | 79.8 ± 11.4 | 75.8 ± 10.6 | 80.0 ± 11.1 | 85.9 ± 11.8 | |
| Body Mass Index, kg/m^2^ (IQR) | 26.2 (4.2) | 25.7 (3.6) | 26.2 (4.2) | 26.9 (4.9) | |
| Total cholesterol, mmol/L | 5.70 ± 1.13 | 5.52 ± 1.09 | 5.71 ± 1.13 | 5.94 ± 1.20 | |
| Smoking, n (%) | 5544 (26%) | 699 (16%) | 4032 (27%) | 813 (35%) | |
| CVD ^a^, n (%) | 1007 (5%) | 335 (8%) | 601 (4%) | 71 (3%) | |
| Hypertension ^b^, n (%) | 8722 (40%) | 1356 (31%) | 5967 (40%) | 1399 (60%) | |
| Diabetes ^c^, n (%) | 565 (3%) | 87 (2%) | 375 (2%) | 103 (4%) | |
| Physical activity level |  |  |  |  | |
| Inactive, n (%) | 2526 (13%) | 382 (10%) | 1814 (13%) | 330 (16%) | |
| Low, n (%) | 3907 (20%) | 574 (14%) | 2830 (21%) | 503 (24%) | |
| Moderate, (%) | 4959 (25%) | 908 (23%) | 3477 (26%) | 574 (28%) | |
| High, (%) | 8224 (42%) | 2157 (54%) | 5409 (40%) | 658 (32%) | |
| Estimated VO_2peak_ ^d^, mL·min^−1^ | 38.1 ± 6.8 | 41.2 ± 6.6 | 37.9 ± 6.5 | 33.5 ± 6.4 | |
| Education |  |  |  |  | |
| Low (7-10 years primary school), n (%) | 3761 (17%) | 643 (15%) | 2612 (18%) | 506 (22%) | |
| Middle (1-2 years high school), n (%) | 11291 (52%) | 2103 (49%) | 7949 (53%) | 1239 (54%) | |
| High (University), n (%) | 6473 (30%) | 1558 (36%) | 4360 (29%) | 555 (24%) | |
| Values are presented as means (± SD), No. (percentages) or median (interquartile range). ^a^ CVD: self-reported stroke, angina pectoris, myocardial infarction, use of antiarrhythmic agents. ^b^ Hypertension: systolic blood pressure (SBP) ≥ 140 mmHg, diastolic blood pressure (DBP) ≥ 90 mmHg or use of medication that influences heart rate or blood pressure. ^c^ Diabetes: non-fasting serum glucose ≥11.1 mmol/L, HbA1c ≥11.1 mmol/L or self-reported diabetes in questionnaire. ^d^ VO_2peak_: estimated peak oxygen consumption. | | | | |  |

| **Supplementary data, Table S3.** Change in resting heart rate and heart failure risk after excluding 59 participants with non-valid HF. | | | | | |
| --- | --- | --- | --- | --- | --- |
|  | Per 10-bpm decreased RHR | Per 10-bpm increased RHR | Maintained RHR  (± 12 bpm) | Decreased RHR  (<-12 bpm) | Increased RHR  (> 12 bpm) |
| Women | 14 851 | 11 113 | 19 216 | 4 413 | 2 335 |
| Person-years | 185 654 | 134 468 | 238 208 | 54 879 | 27 036 |
| No. heart failure | 786 | 5554 | 912 | 282 | 146 |
| Rate/1000 person-years (95% CI) | 4.23 (3.95-4.54) | 4.12 (3.79-4.48) | 3.83 (3.59-4.09) | 5.14 (4.57-5.77) | 5.40 (4.59-6.35) |
| Hazard ratio |  |  |  |  |  |
| Model 1 | 1.13 (1.04-1.22) | 1.20 (1.08-1.34) | 1.00 (ref.) | 1.24 (1.08-1.41) | 1.27 (1.07-1.51) |
| Model 2 | 1.10 (1.00-1.20) | 1.16 (1.04-1.29) | 1.00 (ref.) | 1.12 (0.97-1.30) | 1.18 (0.99-1.41) |
| Model 3 | 1.06 (0.97-1.16) | 1.15 (1.04-1.28) | 1.00 (ref.) | 1.09 (0.94-1.26) | 1.16 (0.97-1.39) |
| Model 4 | 1.04 (0.95-1.14) | 1.15 (1.03-1.28) | 1.00 (ref.) | 1.03 (0.89-1.20) | 1.17 (0.98-1.40) |
| Men | 11 494 | 10 195 | 16 054 | 3 190 | 2 445 |
| Person-years | 141 302 | 124 706 | 198 328 | 38 700 | 28 980 |
| No. heart failure | 833 | 648 | 1033 | 271 | 177 |
| Rate/1000 person-years (95% CI) | 5.90 (5.51-6.31) | 5.20 (4.81-5.61) | 5.21 (4.90-5.54) | 7.00 (6.22-7.89) | 6.11 (5.27-7.08) |
| Hazard ratio |  |  |  |  |  |
| Model 1 | 1.11 (1.03-1.20) | 1.15 (1.05-1.26) | 1.00 (ref.) | 1.14 (1.00-1.30) | 1.19 (1.02-1.40) |
| Model 2 | 1.05 (0.96-1.15) | 1.14 (1.04-1.25) | 1.00 (ref.) | 1.01 (0.87-1.18) | 1.15 (0.97-1.35) |
| Model 3 | 1.04 (0.95-1.13) | 1.12 (1.02-1.23) | 1.00 (ref.) | 0.99 (0.85-1.15) | 1.10 (0.93-1.29) |
| Model 4 | 1.00 (0.91-1.10) | 1.10 (1.00-1.21) | 1.00 (ref.) | 0.93 (0.80-1.08) | 1.08 (0.92-1.27) |
| Number of person-years and heart failures, rate/1000 person years (95% confidence interval (CI)), and hazard ratios (95% CI) for all-cause heart failure in relation to change in resting heart rate (beats per minute (bpm), n = 47 653 (54% women)). Model 1: adjusted for age. Model 2: adjusted for age, physical activity, body mass index, smoking and baseline RHR. Model 3: adjusted for Model 2 and hypertension (systolic blood pressure ≥ 140 mmHg, diastolic blood pressure ≥ 90 mmHg or use of antihypertensive medication), diabetes (non-fasting serum glucose ≥11.1 mmol/L, HbA1c ≥11.1 mmol/L, or self-reported), education and total cholesterol. Model 4: adjusted for Model 3, atrial fibrillation and time-updated self-reported history of stroke, angina pectoris, myocardial infarction and use of medication that influences heart rate. | | | | | |

| **Supplementary data, Table S4.** Baseline RHR and heart failure risk | | | | |
| --- | --- | --- | --- | --- |
|  | Per 10-bpm higher baseline RHR^a^ | Normal RHR  (60-84 bpm) | Low RHR  (< 60 bpm) | High RHR  (≥ 85 bpm) |
| Women |  |  |  |  |
| No. participants (No.) | 23 819 | 19 305 | 2 427 | 4 261 |
| Person-years (years) | 293 478 | 30 111 | 238 475 | 517 46 |
| No. heart failure (No.) | 1 246 | 131 | 969 | 269 |
| Rate/1000 person-years (95% CI) | 4.25 (4.02-4.49) | 4.35 (3.67-5.16) | 4.06 (3.82-4.33) | 5.20 (4.61-5.86) |
| Hazard ratio |  |  |  |  |
| Model 1 | 1.09 (1.04-1.15) | 1.00 (ref.) | 1.03 (0.86-1.24) | 1.32 (1.15-1.51) |
| Model 2 | 1.05 (1.00-1.11) | 1.00 (ref.) | 1.06 (0.89-1.28) | 1.22 (1.07-1.40) |
| Model 3 | 1.04 (0.98-1.09) | 1.00 (ref.) | 1.06 (0.88-1.27) | 1.18 (1.03-1.35) |
| Model 4 | 1.04 (0.99-1.10) | 1.00 (ref.) | 1.02 (0.85-1.23) | 1.19 (1.03-1.36) |
| Men |  |  |  |  |
| No. participants | 17 681 | 15 058 | 4 332 | 2 329 |
| Person-years | 216 213 | 53 670 | 185 107 | 27 427 |
| No. heart failure | 1 222 | 305 | 996 | 210 |
| Rate/1000 person-years (95% CI) | 5.65 (5.34-5.98) | 5.68 (5.08-6.36) | 5.38 (5.06-5.73) | 7.66 (6.69-8.77) |
| Hazard ratio |  |  |  |  |
| Model 1 | 1.15 (1.09-1.21) | 1.00 (ref.) | 0.95 (0.84-1.08) | 1.35 (1.16-1.56) |
| Model 2 | 1.10 (1.04-1.16) | 1.00 (ref.) | 1.05 (0.92-1.20) | 1.22 (1.05-1.42) |
| Model 3 | 1.08 (1.03-1.14) | 1.00 (ref.) | 1.06 (0.94-1.21) | 1.19 (1.03-1.39) |
| Model 4 | 1.09 (1.04-1.15) | 1.00 (ref.) | 1.00 (0.87-1.14) | 1.23 (1.06-1.43) |
| Number of person-years and heart failures, rate/1000 person years (95% confidence interval (CI)), and hazard ratios (95% CI) for all-cause heart failure in relation to baseline resting heart rate (RHR, beats per minute (bpm)), n = 47 712 (53% women) at baseline. Model 1: adjusted for age. Model 2: adjusted for age, physical activity, body mass index and smoking. Model 3: adjusted for Model 2 and hypertension (systolic blood pressure ≥ 140 mmHg, diastolic blood pressure ≥ 90 mmHg or use of antihypertensive medication), diabetes (non-fasting serum glucose ≥11.1 mmol/L, HbA1c ≥11.1 mmol/L, or self-reported), education and total cholesterol. Model 4: adjusted for Model 3, atrial fibrillation and time-updated self-reported history of stroke, angina pectoris, myocardial infarction and use of medication that influences heart rate. ^a^ Starting from 60 bpm | | | | |
